# Taxonomic Revision of *Ampelomyces* Strains *(Dothideomycetes*, *Pleosporales)* from All-Russian Collection of Microorganisms

**DOI:** 10.3390/jof12080558

**Published:** 2026-07-30

**Authors:** Nataliya Ivanushkina, Anastasia Danilogorskaya, Galina Kochkina

**Affiliations:** All-Russian Collection of Microorganisms (VKM), G.K. Skryabin Institute of Biochemistry and Physiology of Microorganisms (IBPM RAS), Pushchino Scientific Center for Biological Research of the Russian Academy of Sciences (PSCBR RAS), 142290 Pushchino, Russia; ivanushkina@pbcras.ru (N.I.); danilogorskaya@pbcras.ru (A.D.)

**Keywords:** *Ampelomyces*, phylogeny, hyperparasitism, plant protection

## Abstract

Fungi of the genus *Ampelomyces* are among the most significant hyperparasites used for biological control of phytopathogenic fungi, particularly those causing powdery mildew on various plants. Most species of this genus were described by the Soviet scientist O. Rudakov (1979), 13 original strains of which, including several type cultures, are deposited in the All-Russian Collection of Microorganisms (VKM). These strains were studied for their morphological characteristics and multilocus phylogenetic analysis (ITS, LSU, tub2, and rpb2). However, none of them are *Ampelomyces*. Based on the results obtained, it was established that all studied strains belong to other taxa of the order *Pleosporales*: *Didymella glomerata*, *D. pomorum*, *Nothophoma brennandiae*, *N. quercina*, *N. spiraeae.* The species *Ampelomyces artemisiae*, *A. heraclei*, *A. polygoni*, *A. ulicis*, and *A. uncinulae*, for which the material type was studied, should be the heterotypic (taxonomic) synonyms of the taxon *Didymella glomerata*. Historical experimental data confirming hyperparasitism are provided for nine of the studied strains.

## 1. Introduction

Fungi of the genus *Ampelomyces* (*Pezizomycotina*, *Dothideomycetes*, *Pleosporales*, *Phaeosphaeriaceae*) are the oldest biological antagonists of powdery mildew fungi (fungi of the family *Erysiphaceae*), which infect agricultural plants across the globe. Fungi of the genus *Ampelomyces* were among the first mycophilic fungi to be thoroughly studied [1]. This genus, with its enormous practical significance, is currently the subject of intense study by mycologists. The fungi described as *Ampelomyces* have been studied by many scientists, who have established that the early stage of mycoparasitism by these fungi appears to be biotrophic; however, subsequently, the infected parasite cytoplasm begins to die off, and the fungal interaction progresses to a necrotrophic stage. The hyperparasitic fungus does not produce toxins; however, it does suppress sporulation [2]. Thus, they were among the first micromycetes used for biological control of plant-parasitic fungi. Various biofungicides like them have been developed and patented based on their environmentally friendly and cost-effective nature [3,4]. Overall, *Ampelomyces* strains have been reported in association with more than 65 fungal species from eight genera of the *Erysiphaceae* family worldwide [5].

According to Mycobank (https://www.mycobank.org/) on 01 April 2026, the genus *Ampelomyces* currently contains 18 legitimate species, 16 of which were described by the renowned Soviet mycologist O. Rudakov (1979) in the journal “Mycology and Phytopathology” in Russian [6] but are not widely accessible to specialists. Some strains were deposited in the All-Russian Collection of Microorganisms (VKM), where they have been stored since the 1980s. However, species of this genus have been described solely based on phenotypic characteristics, considering the specialization of hyperparasites to the fungal host species and, often, the host plants. Our thorough analysis of the macro- and micromorphological characteristics of species revealed that the distinctions among them are unclear, and the association of species with both parasitic fungi and host plants is quite broad. At one time, this even served as the basis for combining all powdery mildew hyperparasites into a single species—*Ampelomyces quisqualis*. However, most specialists did not accept this, and various species of this genus exist. The primary criteria for defining species boundaries were the shape and size of conidia, specialization of the host fungi, and the cultural–morphological characteristics of colonies on nutrient media [6].

In Mycobank, all species described by Rudakov are currently transferred to the genus *Cicinnobolus*, except for *Ampelomyces quercinus* and *Ampelomyces parasiticus*, which are now named *Nothophoma quercina* and *Phyllosticta parasitica*, respectively. This is likely because until the mid-1970s, de Bary’s proposed name *Cicinnobolus* was widely used for powdery mildew hyperparasites, and the description of the genus *Ampelomyces* was incomplete. Although some studies still classify powdery mildew hyperparasites as members of the genus *Ampelomyces* based solely on morphological characteristics [7], studies have emerged showing that formally described species do not match the phylogenetic clades derived based on DNA sequence analysis. Therefore, a taxonomic revision of the genus is necessary [5]. Numerous sequences of fungi of the genus *Ampelomyces* are publicly available in the NCBI genome database, but it is unknown whether they actually belong to this genus [8]. Importantly, there are assumptions that some *Didymella* or *Phoma* strains were misidentified and deposited under the name *Ampelomyces* in the world’s largest mycological collections. This poses a serious challenge to further research based on information from open databases [9]. The VKM collection is no exception.

The high practical value of *Ampelomyces* genus members and the need to study their phylogenetic relationships served as the basis for this taxonomic review of 13 strains from this genus in the VKM. Within the framework of a study based on polyphasic taxonomic methods, the type and syntype strains of species described by O. L. Rudakov in 1979 were studied [6].

## 2. Materials and Methods

### 2.1. Morphological Studies

Thirteen *Ampelomyces* strains from the VKM fungi collection were studied (Table 1). Cultures were cultivated on malt extract agar (MEA), oatmeal agar (OA), and potato dextrose agar (PDA) at 25 °C. Colony diameters were measured after 7 days of incubation, and colony characteristics were noted. The color of colonies was determined on OA according to alphanumeric codes of Kornerup and Wanscher (1978) [10]. Morphological observations of reproductive structures were made from cultures grown on OA. Pycnidia and conidia (50 for each strain) were measured under a microscope at 400× magnification.

### 2.2. DNA Extraction, PCR Amplification, and Sequencing

The internal transcribed spacer (ITS), large subunit (LSU) of ribosomal DNA, partial DNA-directed RNA polymerase II subunit (rpb2), and β-tubulin (tub2) genes were amplified and sequenced for the studied strains. The primers ITS5/ITS4 [11] and LR0R [12]/LR5 [13] were used to amplify ITS and LSU regions, while the primers Btub2Fd/Btub4Rd [14] and RPB2-5F/RPB2-7CR [15] were used to amplify the partial tub2 and rpb2 genes, respectively. The amplification reactions were performed in a total volume of 25 μL, including a 2.5× reaction mixture for PCR from Syntol (https://www.syntol.ru (accessed on 12 January 2026); cat. N M-428) at 10 μL, MgCl_2_ (25 μM) at 1.5 μL, forward primer (10 μM) at 1 μL and reverse primer (10 μM) at 1 μL, dd H_2_O at 10.5 μL and a resulting template DNA solution of 1 μL. PCR was carried out according to the [16] protocol with annealing temperatures for the primer pairs LR0R/LR5 and Btub2Fd/Btub4Rd selected experimentally. The final PCR conditions for the amplification were as follows: 95 °C for 3 min; 34 cycles at 95 °C for 15 s; 53 °C for 15 s (ITS5/ITS4) or 55 °C for 15 s (LR0R/LR5) or 58 °C (RPB2-5F/RPB2-7CR) or 62 °C for 15 s (Btub2Fd/Btub4Rd), and 72 °C for 1 min; and a final elongation for 5 min at 72 °C. PCR amplification products were stained with SYBR Green I and checked using electrophoresis in 1% agarose gel.

Enzymatic purification of amplicons and their subsequent sequencing using Sanger’s method were performed at Eurogen (https://www.evrogen.ru/ (accessed on 1 April 2026)). The results were compared with sequences in the NCBI BLAST database (see Supplementary Material). The nucleotide sequences of the ITS, LSU, rpb2, and tub2 genes obtained were deposited in the GenBank database with corresponding accession numbers.

### 2.3. Phylogenetic Analysis

Sequences were inspected using the Bioedit sequence alignment editor v. 7.0.0 (https://bioedit.software.informer.com/7.0/ (accessed on 1 April 2026)) and aligned with MEGA 6.06. The alignments were concatenated using the Microsoft Excel 2010 text formula “Concatenate”. A multilocus phylogenetic analysis was based on the combined ITS, LSU, rpb2, and tub2 sequences for all strains studied, all species currently accepted in the genus *Nothophoma* (Qian Chen & L. Cai) [17], and those studied that were closest to the genus *Didymella* Saccardo [17,18] for which nucleotide sequences were available. The tree was rooted using the ex-type *Phoma herbarum* (CBS 615.75). Sequences of representative *Didymellaceae* strains and type species were obtained from GenBank (Table S1—Supplementary Materials).

Phylogenetic analysis of combined aligned data was performed using both maximum likelihood (ML) and Bayesian tree inference. Maximum likelihood analysis was conducted in IQ-TREE [19] with default parameters. Modeltest in IQ-TREE was used to determine the most suitable nucleotide substitution model for nucleotide datasets according to the Bayesian information criterion (BIC), TNe + I + G4. The combined dataset was analyzed as a single unpartitioned matrix; ModelFinder identified TNe + I + G4 as the optimal overall model. Bootstrap values with 1000 replicates were calculated for tree branches. Bayesian inference was performed in MrBayes 3.2.1 using a Markov chain Monte Carlo (MCMC) sampling method. The analysis was performed using two independent runs, each utilizing four chains (one cold and three heated), under default software settings unless otherwise specified. Sampling was performed every 100 generations, and the chains were run continuously until the average standard deviation of split frequencies dropped below the strict convergence threshold of 0.01. A standard 25% burn-in fraction was applied to discard trees sampled prior to stationarity. Maximum likelihood phylograms were used to represent data with both bootstrap values ≥80% and/or posterior probabilities ≥95%. Trees were visualized on the interactive tree of life (iTOL) v. 3 [20].

## 3. Results

### 3.1. Morphological Characteristics

Table 2 shows the colony sizes on different media and individual phenotypic characteristics of cultures on OA medium.

All strains exhibited significant similarity in growth rate and the morphological characteristics of colonies for different media. Colonies of all strains, in the case of abundant aerial mycelium formation, were elevated, with a woolly to flaky surface. In the absence of aerial mycelium, colonies were flat and only slightly woolly, with a large number of superficial pycnidia. Three strains were sterile on all media tested (VKM F-2782, VKM F-2799, and VKM F-5158). The remaining cultures showed pycnidial sporulation. Pycnidia are light-to-dark brown, thin-walled, and consist of polygonal cells. Conidiogenous cells are enteroblastic phialides; conidia are unicellular, hyaline, oval-to-elliptical, and smooth-walled. Cultures of VKM F-2800 and VKM F-5157, in addition to conidia, formed chains of *Alternaria*-like chlamydospores measuring 22–45 × 12–18 µm.

All cultures with sporulation were divided into two groups. The first group had eight cultures—VKM F-2758, VKM F-2768, VKM F-2794, VKM F-2797, VKM F-2800, VKM F-2839, VKM F-5157, VKM F-5160—with similar-sized pycnidia (aver. 71–162 × 60–120 µm) and differently sized biguttulate thin-walled conidia (aver. 3.8–6.1 × 1.9–2.7 μm). Two cultures—VKM F-5159 and VKM F-5161—had significantly larger (2.5 times) pycnidia, wider conidia than those of the first group of strains, thick walls, and no guttulate (aver. 4.3–6.5 × 3.1–4.1 μm).

**Figure 1:**
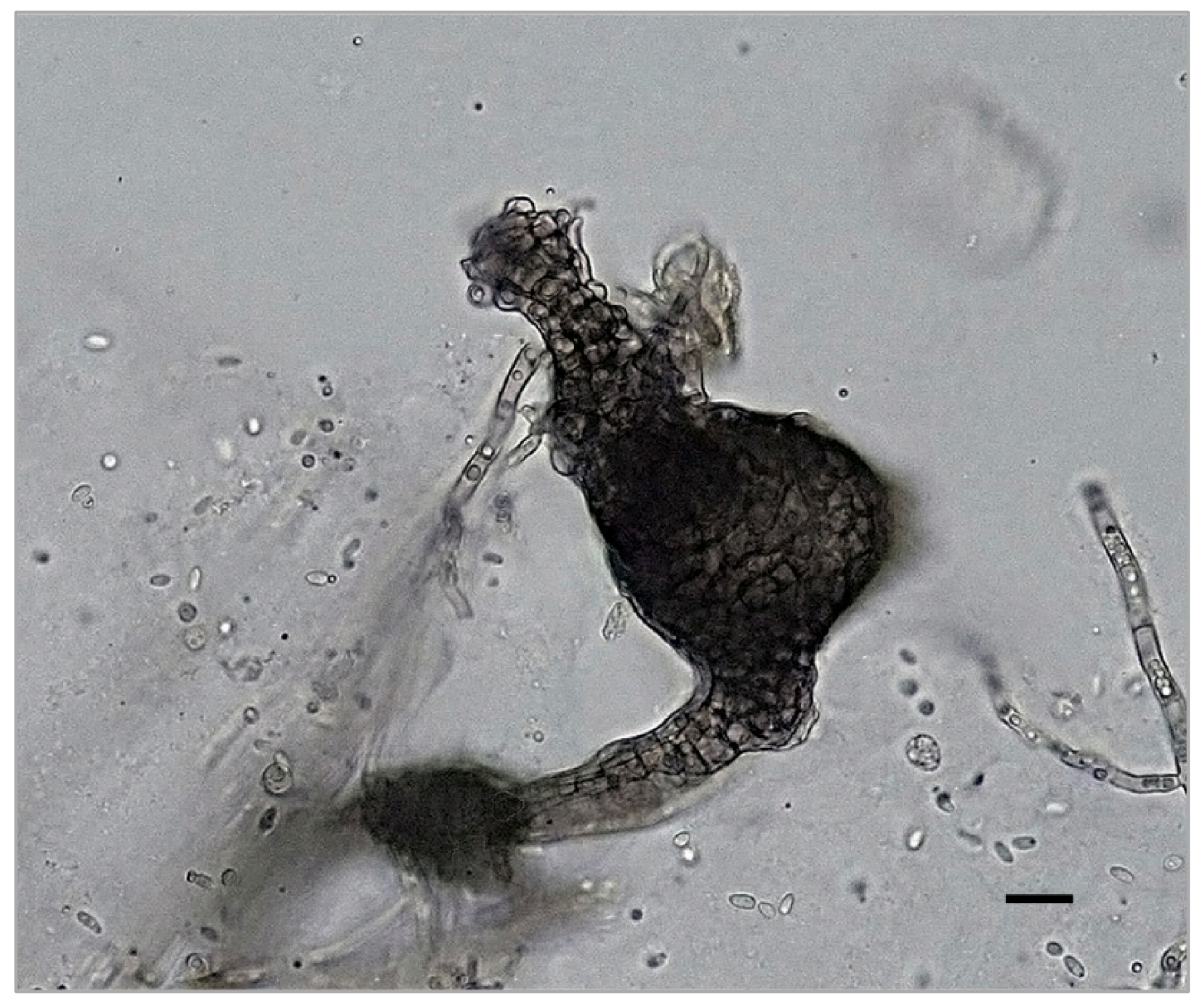
Pycnidium on the stalk of *Didymella glomerata* VKM F-2800. Scale bar 10 μm.

It should be noted that the morphological characteristics of the studied cultures are very similar to those of the *Phoma* sensu lato species complex. In particular, when dividing the genera *Ampelomyces* and *Phoma* by morphology, one of the main criteria is the immersion of pycnidia in the mycelium (*Phoma*) or their superficial location (*Ampelomyces*) [21]. However, most often this relates to the morphology of cultures on natural substrates, whether plant leaves or fungi. In vitro, especially after several passages, morphology can change, including the number and arrangement of pycnidia. Figure 1 shows a pycnidial conidiomata with a long stalk of the culture VKM F-2800, later identified as *Didymella glomerata*.

### 3.2. Multilocus Phylogenetic Analysis

**Figure 2:**
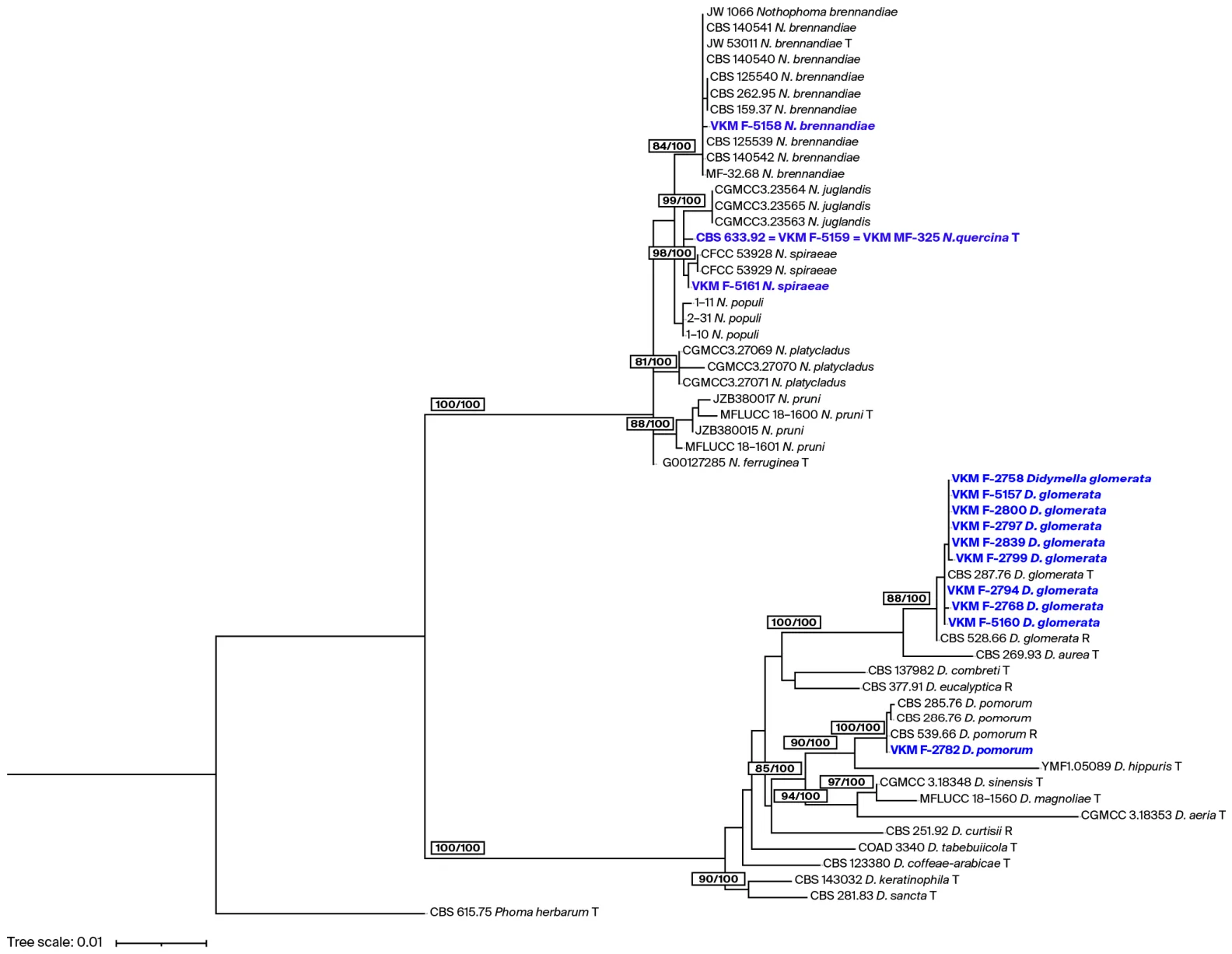
Phylogenetic tree of *Didymella* and *Nothophoma* species and strains constructed based on maximum likelihood analysis using the combined ITS, LSU, rpb2, and tub2 datasets. Maximum likelihood bootstrap support values (MLBS ≥ 80%) and Bayesian posterior probabilities (BPP ≥ 95%) are indicated at nodes (MLBS/BPP). Studied strains are shown in blue, and type and representative strains are marked with T or R, respectively, and are presented in Table S1.

The results of multilocus phylogenetic analysis based on the ITS, LSU, rpb2, and tub2 sequences are presented in Table 3 and Figure 2.

All strains were closely related to representatives of phoma-like fungi of the family *Didymellaceae* and were also in the order *Pleosporales*. It was clear that none of the studied strains belonged to the genus *Ampelomyces* (order *Pleosporales*, family *Phaeosphaeriaceae*). We also compared the studied strains with the representative cultures of the genus *Ampelomyces*, and phylogenomic differences were confirmed (Table S2).

## 4. Discussion

Several decades ago, when studying hyperparasites on powdery mildew fungi, the presence of intracellular pycnidia automatically indicated the presence of fungi of the genus *Ampelomyces*, and studies mainly focused on determining the natural occurrence of hyperparasites and the intensity of mycoparasitism [22]. Their separation from representatives of phoma-like fungi based on cultural–morphological characteristics is difficult.

Many fungi have distinct phenotypic characteristics that allow for a fairly accurate identification of their taxon, at least at the genus level. This is not the case with *Ampelomyces*. The description of species in the genus *Ampelomyces* by Rudakov (1979) [6] was carried out based on phenotypic characteristics on nutrient media immediately after isolation. Since the pycnidia of each strain were found to be highly variable, the main criteria for distinguishing species were colony growth characteristics and conidia morphology. A detailed study of the morphology of the same strains after half a century of storage in the VKM revealed that differences in morphology exist; however, in some cases, they are within the range of intraspecific variability (Table 2).

*Phoma* is the most widespread genus in the order *Pleosporales*, comprising over 3000 species, and over 100 species are known as phytopathogens. This genus is polyphyletic and taxonomically controversial, with unclear boundaries. *Phoma* fungi are commonly associated with devastating diseases in economically important agricultural crops. These fungi can produce various phytotoxins that impair photosynthesis and cause electrolyte leakage from plant cells [23]. However, hyperparasitism has been infrequently reported regarding its representativeness in this genus. For example, *P. glomerata* (tm. *Didymella glomerata*) has been shown to initiate mycoparasitism in relation to *Microsphaera penicillata*, which causes leaf disease in sycamore trees [24]. Strains of *Phoma* sp. have been found to be hyperparasitic in relation to the basidiomycete *Puccinia melanocephala*, the causal agent of sugarcane rust in Cuba [25]. However, all these studies were conducted before the genomic era, when fungi were described solely based on morphological characteristics, so there is uncertainty about the correct taxonomic classification of mycophilic fungi.

A recent phylogenetic study of a wide range of fungi in the family *Didymellaceae*, order *Pleosporales*, conducted multilocus phylogenetic analysis based on ITS, LSU, rpb2, and tub2 sequences, taking into account morphological differences. This analysis not only clarified the phylogenetic relationships and established boundaries [26] of these species, but also determined their differences from fungi in the family *Phaeosphaeriaceae*, in which genus *Ampelomyces* is included. Species morphologically similar to fungi of the genus *Phoma* were found in at least six families of the order *Pleosporales*. Furthermore, there is evidence that phoma-like fungi are distributed across 20 families of six orders of ascomycetous fungi [18].

About a thousand sequences of fungi of the genus *Ampelomyces* have been deposited in the NCBI database. However, when compared with VKM fungi sequences, they were not closely related. Since there is no certainty that all deposited sequences belong specifically to fungi of the genus *Ampelomyces*, for a detailed comparison, we selected sequences from strains in the largest global collections: CBS (Netherlands), ATCC (USA), and DSM (Germany). It turned out that 34 sequences could be compared at the ITS and LSU loci. None of them showed close similarity to VKM strains (Table S2). The similarity percentages for ITS and LSU were in the range 82.88–95.76% and 92.52–95.57%, respectively. Thus, none of the strains studied belonged to the genus *Ampelomyces*.

The results of phylogenetic analysis for most VKM strains exhibited similarity to fungi of the genus *Didymella*. The majority of strains were assigned to *Didymella glomerata* (VKM F-2758, VKM F-2768, VKM F-2794, VKM F-2797, VKM F-2799, VKM F-2800, VKM F-2839, VKM F-5157, and VKM F-5160) (Table 2). As shown in the table, spore-bearing strains of this group have similar micromorphology in their conidiogenous apparatus. Their cultural–morphological characteristics are also similar and correspond to the description of *Didymella glomerata* based on a study, among other cultures, of the representative culture CBS 528.66 in vitro [27], as shown in Table 4.

Representatives of *D. glomerata* are considered widespread soil saprotrophs. There are several reports of these fungi on inorganic substrates, including asbestos, cement, and paint [26]. Their geographic distribution is large: in Russia, strains of this species have been detected on *Apiaceae*, *Liliaceae*, *Rosaceae*, and *Salicaceae* plants [18]. The strains studied in our work—most of which belong to the species *D. glomerata*—were subjected to experimental testing to determine their parasitic activity against powdery mildew pathogens after isolation from the natural environment by Rudakov. Cucumber (*Cucumis sativus*) leaves were infected with the phytopathogenic fungi *Botrytis cinerea* and *Erysiphe cichoracearum* (current name: *Golovinomyces cichoracearum*). Afterward, conidial suspensions of hyperparasites were applied dropwise to the phytopathogenic fungi, and the inhibitory effect was observed. Spores germinated, and hyphae entwined with the host fungal mycelium and penetrated deep into the cells [1]. The strains listed in Table 1 were found to be parasitic based on Rudakov’s data.

The strain of VKM F-2782, defined as *Ampelomyces quisqualis*, was classified as *Didymella pomorum* based on phylogenetic analysis. Unfortunately, this strain was sterile and did not form conidiogenic structures on any of the media we used. However, the cultural–morphological properties of this strain [31] do not contradict its assignment to the species via molecular genetic analysis (Table 4). It was found that similarity to the representative culture of *Didymella pomorum* CBS 539.66 is 100% (LSU and rpb2) and 99.79% (ITS) (Table 4). Representatives of this species are opportunistic pathogens that prefer a temperate climate and are often found in soil and plant seeds [27], as well as plants of various families, including Russia. Strain VKM F-2782 also exhibited parasitic properties in laboratory experiments by O. Rudakov (Table 1).

**Figure 3:**
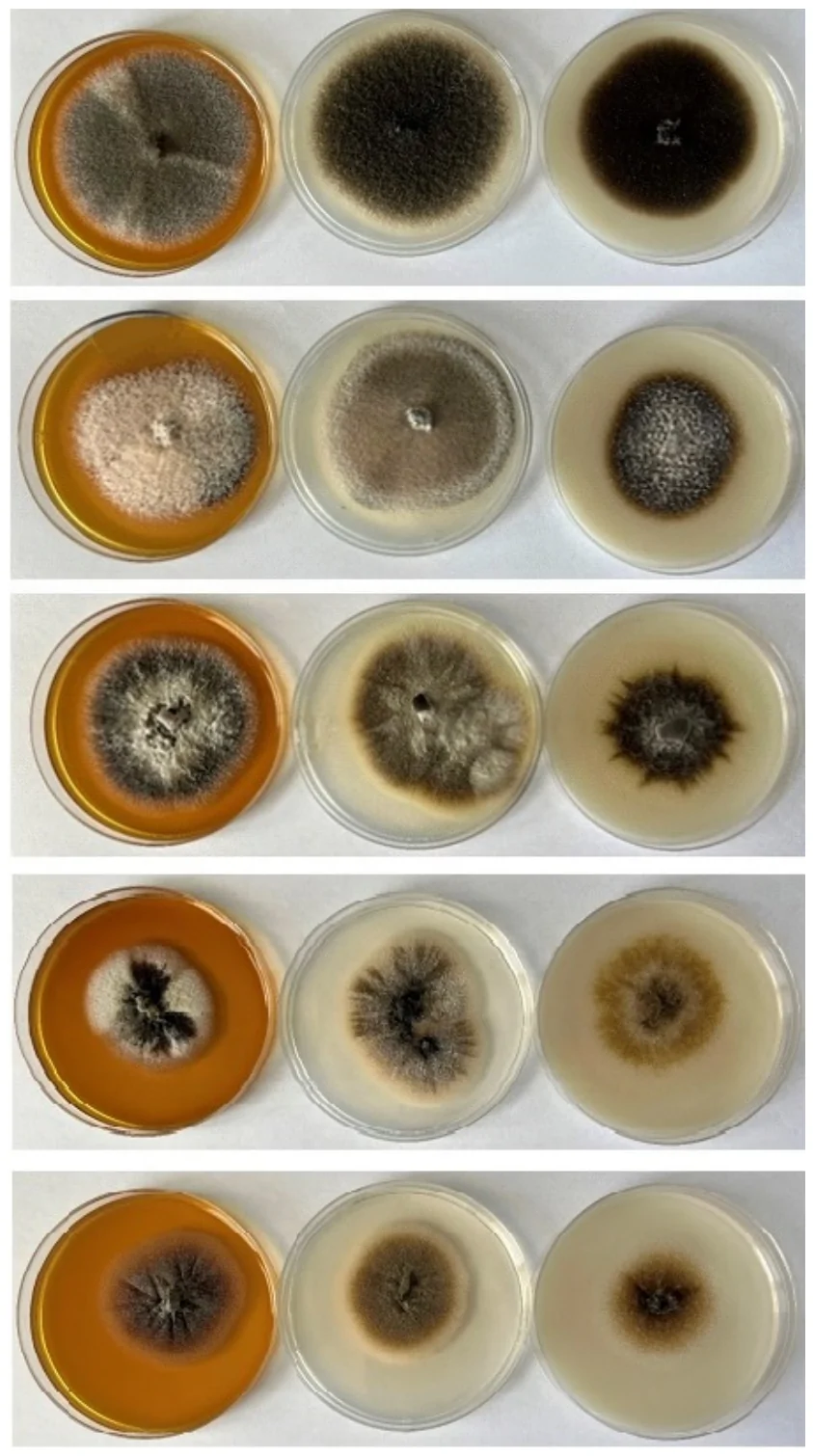
Colony morphology in 7 days on MEA, PDA and OA (from **left** to **right**) of strains (from **top** to **bottom**): VKM F-2800 *Didymella glomerata* (previous name *A. humuli*); VKM F-2782 *Didymella pomorum* (previous name *A. quisqualis)*; VKM F-5158 *Nothophoma brennandiae* (previous name *A. quercinus*); VKM F-5159 *Nothophoma quercina* (previous name *A. quercinus*); VKM F-5161 *Nothophoma spiraeae* (previous name *Ampelomyces* sp.).

**Figure 4:**
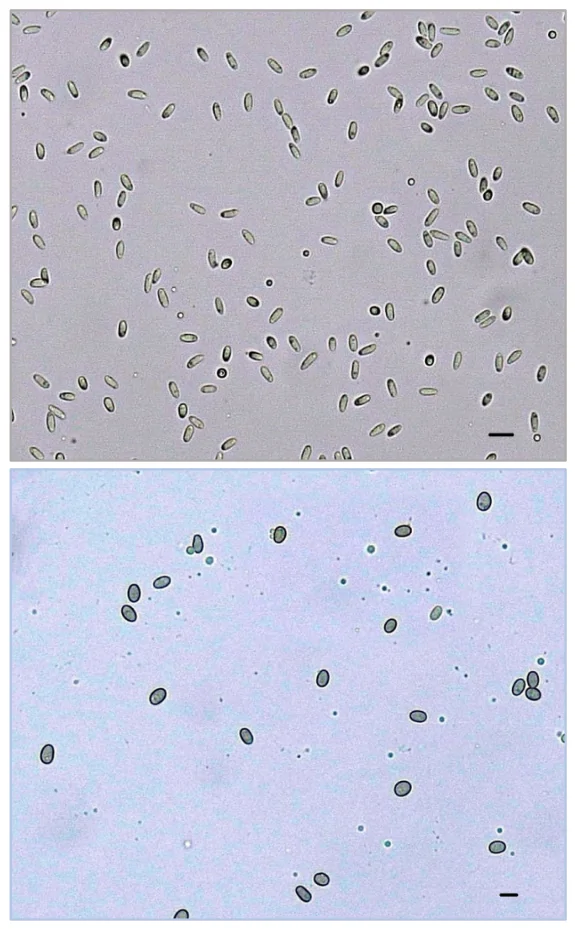
Conidia of *Didymella glomerata* VKM F-2800 (**top**) and *Nothophoma spiraeae* VKM F-5161 (**bottom**). Scale bar 5 μm.

The division of strains into two groups based on morphological characteristics (Table 2) was also reflected in the results of multilocus testing. Two strains, VKM F-5159 and VKM F-5161, exhibited significant differences in micromorphology compared to other strains studied (Figure 3 and Figure 4). The larger pycnidia and ovoid, thick-walled conidia with a length-to-width ratio of 1.4 distinguished them from strains classified as belonging to the genus *Didymella*. These had a length-to-width ratio of 2.2 and thin-walled, ellipsoidal conidia. These strains, as well as a sterile strain of VKM F-5158, were assigned to the genus *Nothophoma*.

This genus was described in 2015 [32] based on a multilocus phylogenetic analysis of a large number of strains, attempting to divide the genera into an *Ascochyta–Didymella–Phoma* complex. As a result, nine new genera were described with a high level of accuracy, including the genus *Nothophoma*. The experimental data classified this genus as a member of the family *Didymellaceae*. The strain VKM F-5159, originally identified as *Ampelomyces quisqualis* and later transferred to the CBS collection (CBS 633.92), has been included in several phylogenetic studies and was initially considered *Phoma fructicola* [29]. However, according to phylogenetic analysis, it was then classified in the *Nothophoma* cluster as a type strain of the species *Nothophoma quercina* [32]. The replica of this strain stored in VKM was assigned to this species.

The strain VKM F-5158, defined as *Ampelomyces quercinus*, was found to be a representative of the species *Nothophoma brennandiae*, first described in strains isolated from soil samples in the Netherlands [28]. Subsequently, these fungi were found in Europe associated with plants of the families *Betulaceae*, *Oleaceae*, *Rosaceae* and *Ulmaceae*, and in Canada in domestic dust [17]. The similarity of the strain VKM F-5158 to the holotype culture of *Nothophoma brennandiae* JW53011 (CBS 145912) is 100% (ITS, LSU and rpb2) and 99.69% (tub2). A strain stored in VKM was extracted from the powdery mildew of an oak tree.

Strain VKM F-5161 was assigned to the species *Nothophoma spiraeae*. This is confirmed by the results of molecular genetic analysis and is not contradicted by the morphology of this strain or its comparison with the morphology of this species’ type strain CFCC 53928 [30] (Table 4).

Thus, studying the type of material of species *Ampelomyces artemisiae*, *A. heraclei*, *A. polygoni*, *A. ulicis*, and *A. uncinulae*, as described by O. Rudakov [6], showed that they are heterotypic (taxonomic) synonyms of the taxon *Didymella glomerata*.

**Taxonomy.**

*Didymella glomerata* (Corda) Q. Chen & L. Cai 2015

= *Ampelomyces artemisiae* (Voglino 1905) Rudakov 1979

= *Ampelomyces heraclei* (Dejeva 1967) Rudakov 1979

= *Ampelomyces polygoni* (Potebnia 1907) Rudakov 1979

= *Ampelomyces ulicis* (J.F. Adams 1907) Rudakov 1979

= *Ampelomyces uncinulae* (Fautrey 1893) Rudakov 1979

***Ampelomyces artemisiae*** (Voglino) Rudakov 1979

*Typus*: Republic of Moldova, fungus, *Golovinomyces cichoracearum* (syn. *Erysiphe cichoracearum*) on *Acacia* sp., MF-332 (VKM F-2794).

The type strain corresponds morphologically (Table 2) and phylogenetically (Figure 2) to *Phoma glomerata*, which justifies the recognition of this species as a heterotypic synonym of *Phoma glomerata*.

***Ampelomyces heraclei*** (Dejeva) Rudakov 1979

*Typus*: Republic of Moldova, fungus, *Plasmopara viticola*, MF-245 (VKM F-2768).

The type strain corresponds morphologically (Table 2) and phylogenetically (Figure 2) to *Phoma glomerata*, which justifies the recognition of this species as a heterotypic synonym of *Phoma glomerata*.

***Ampelomyces polygoni*** (Potebnia) Rudakov 1979

*Syntypus*: Krasnodar, Russia, fungus, *Erysiphe cruciferarum* on *Polygonum* sp., MF-197 (VKM F-2758).

*Syntypus*: Krasnodar, Russia, fungus, *Erysiphe cruciferarum* on *Polygonum* sp., MF-368 (VKM F-2799).

The syntypes strains correspond morphologically (Table 2) and phylogenetically (Figure 2) to *Phoma glomerata*, which justifies the recognition of this species as a heterotypic synonym of *Phoma glomerata*.

***Ampelomyces ulicis*** (J. Adams) Rudakov 1979

*Typus*: Moscow Region, Russia, fungus, *Erysiphe cruciferarum* on *Convolvulus arvensis*, MF-342 (VKM F-2797).

The type strain corresponds morphologically (Table 2) and phylogenetically (Figure 2) to *Phoma glomerata*, which justifies the recognition of this species as a heterotypic synonym of *Phoma glomerata*.

***Ampelomyces uncinulae*** (Fautrey) Rudakov 1979

*Typus*: Republic of Moldova, fungus, *Podosphaera clandestina* (syn. *Erysiphe clandestina*), MF-484 (VKM F-2839).

The type strain corresponds morphologically (Table 2) and phylogenetically (Figure 2) to *Phoma glomerata*, which justifies the recognition of this species as a heterotypic synonym of *Phoma glomerata*.

For all other species described by Rudakov, the type of material was not preserved, or the species were described based on the study of only herbarium material that has not survived to this day.

Since some strains re-identified here as *Didymella glomerata* exhibited hyperparasitic behavior under the conditions studied by Rudakov, our research suggests that hyperparasitism is possible for a wider range of taxa of the order *Pleosporales*, in addition to *Ampelomyces*.

Hyperparasitic fungi are of great ecological importance, mediating interactions between host plants and primary parasites and playing a significant role in regulating the population sizes of both partners. Certainly, the nature of hyperparasitic relationships needs to be further investigated, and all strains of the genus *Ampelomyces* stored in mycological culture collections should be carefully checked and, if necessary, redescribed.

## Figures and Tables

**Table 1:** List of strains studied in the current work.

Number in Collections	Taxon Name (Received as)	Identification Number ^1^	Status of the Strain ^2^	Substrate (Including Host)
VKM F-2758 ^3^ (ATCC 38608)	*Ampelomyces polygoni*	MF-197	Syntype	fungus, *Erysiphe cruciferarum* on *Polygonum* sp., Krasnodar, Russia
VKM F-2768 ^4^ (ATCC 36804)	*Ampelomyces heraclei*	MF-245	Type	fungus, *Plasmopara viticola*, Republic of Moldova
VKM F-2782 ^3^ (ATCC 36856)	*Ampelomyces quisqualis*	MF-283	-	fungus, *Golovinomyces cichoracearum* (syn. *Erysiphe cichoracearum*), Caucasus area, Russia
VKM F-2794 ^3^ (ATCC 38609)	*Ampelomyces artemisiae*	MF-332	Type	fungus, *Golovinomyces cichoracearum* (syn. *Erysiphe cichoracearum*) on *Acacia* sp., Republic of Moldova
VKM F-2797 ^3^	*Ampelomyces ulicis*	MF-342	Type	fungus, *Erysiphe cruciferarum* on *Convolvulus arvensis*, Republic of Moldova
VKM F-2799 ^3^	*Ampelomyces polygoni*	MF-368	Syntype	fungus, *Erysiphe cruciferarum* on *Polygonum* sp., Krasnodar, Russia
VKM F-2800 ^3^ (ATCC 38616)	*Ampelomyces humuli*	MF-369	-	fungus, *Podosphaera macularis* (syn. *Sphaerotheca macularis*) on *Potentilla* sp., Moscow Region, Russia
VKM F-2839 ^3^ (ATCC 36853)	*Ampelomyces uncinulae*	MF-484	Type	fungus, *Podosphaera clandestina* (syn. *Erysiphe clandestina*), Republic of Moldova
VKM F-5157	*Ampelomyces* sp.	MF-350	-	fungus, powdery mildew on *Polygonum* sp., Astrakhan, Russia
VKM F-5158 ^3^ (ATCC 38614)	*Ampelomyces quercinus*	MF-349	-	fungus, *Erysiphe alphitoides* (syn. *Microsphaera alphitoides*) on *Quercus* sp., Russia
VKM F-5159 (CBS 633.92, ATCC 36786)	*Ampelomyces quercinus*	MF-325	-	fungus, *Golovinomyces cichoracearum* (syn. *Erysiphe cichoracearum*) on *Quercus* sp., Russia
VKM F-5160	*Ampelomyces ulicis*	MF-348	-	fungus, *Erysiphe communis* on *Medicago* sp., Moscow Region, Russia
VKM F-5161	*Ampelomyces* sp.	MF-343	-	fungus, *Golovinomyces cichoracearum* (syn. *Erysiphe cichoracearum*) on *Alnus* sp., Khanka Lake, Far East, Russia

^1^ The number given to the strain when it was isolated from the natural substrates. ^2^ Type strain (or nomenclatural type) is a reference specimen of a fungus to which the name of a particular species is officially associated. Syntype is one of several strains simultaneously designated in the protologue. ^3^ Experimentally studied parasitism on the host *Erysiphe cichoracearum* (current name—*Golovinomyces cichoracearum*) [1]. ^4^ Experimentally studied parasitism on the host *Botrytis cinerea* [1].

**Table 2:** Phenotypic characteristics of the studied strains.

Strain Number	Colony Diameter (cm) After 7 Days on Media			Colony Colours on OA		Size (μm)	
	OA	PDA	MEA	Mycelium	Reverse	Pycnidia	Conidia
VKM F-2758	7.0	7.5	8.0	white	greyish-brown	56–133 × 52–96	3.7–6.0 × 2.0–2.6
VKM F-2768	7.0	7.5	7.5	brownish-grey	brownish-grey	66–173 × 65–149	4.1–7.0 × 1.8–3.2
VKM F-2782	5.0	7.0	7.0	white in center, olive-brown in periphery	greyish-brown	no	no
VKM F-2794	7.0	8.0	7.5	brownish-grey	brownish-grey	69–156 × 65–139	3.9–5.8 × 1.9–2.9
VKM F-2797	6.0	7.3	8.0	greyish-brown	brownish-grey	103–250 × 83–178	4.1–5.8 × 1.9–2,6
VKM F-2799	6.0	5.8	6.5	brown in center, light in periphery	brown	no	no
VKM F-2800	6.5	7.5	7.0	dark-brown	brownish-grey	59–101 × 35–61	3.7–6.1 × 1.8–2.6
VKM F-2839	7.0	8.0	7.5	light-brown	brown	60–131 × 40–82	3.5–6.0 × 1.8–2.7
VKM F-5157	7.0	8.0	7.0	light-brown	brown	45–100 × 42–78	3.7–6.4 × 1.8–2.5
VKM F-5160	5.5	6.5	7.0	white in center, orange-gray in periphery	white	71–165 × 68–152	3.5–6.3 × 2.0–2.8
VKM F-5158	5.5	6.5	6.5	brown	brown	no	no
VKM F-5159	5.0	5.3	4.8	brownish-orange	white	235–474 × 149–329	4.0–6.1 × 3.2–4.2
VKM F-5161	4.8	5.3	5.0	yellowish-brown	dark-blond	155–433 × 144–298	4.6–6.8 × 3.0–4.0

**Table 3:** Results of multilocus phylogenetic analysis of studied strains based on the ITS, 28S, rpb2, and tub2 sequences.

Strain Number	Deposited in VKM as	GenBank Accession Numbers				Identified as
		ITS	tub2	rpb2	LSU	
VKM F-2758	*Ampelomyces polygoni* (Potebnia 1907) Rudakov 1979	-	PX283766	PX283753	*-*	*Didymella glomerata* (Corda 1840) Qian Chen et L. Cai 2015
VKM F-2768	*A. heraclei* (Dejeva 1967) Rudakov 1979	PX289730	PX283767	PX283754	PX289754	*D. glomerata* (Corda 1840) Qian Chen et L. Cai 2015
VKM F-2794	*A. artemisiae* (Voglino 1905) Rudakov 1979	PX289732	PX283768	PX283756	PX289756	*D. glomerata* (Corda 1840) Qian Chen et L. Cai 2015
VKM F-2797	*A. ulicis* (J.F. Adams 1907) Rudakov 1979	PX289733	PX283769	PX283757	PX289757	*D. glomerata* (Corda 1840) Qian Chen et L. Cai 2015
VKM F-2799	*A. polygoni* (Potebnia 1907) Rudakov 1979	PX289734	PX283770	PX283758	PX289758	*D. glomerata* (Corda 1840) Qian Chen et L. Cai 2015
VKM F-2800	*A. humuli* (Fautrey 1890) Rudakov 1979	PX289735	PX283771	PX283759	PX289759	*D. glomerata* (Corda 1840) Qian Chen et L. Cai 2015
VKM F-2839	*A. uncinulae* (Fautrey 1893) Rudakov 1979	PX289736	PX283772	PX283760	PX289760	*D. glomerata* (Corda 1840) Qian Chen et L. Cai 2015
VKM F-5157	*Ampelomyces* sp.	PX275617	PX283773	PX283761	PX275713	*D. glomerata* (Corda 1840) Qian Chen et L. Cai 2015
VKM F-5160	*A. ulicis* (J.F. Adams 1907) Rudakov 1979	PX275620	PX283776	PX283764	PX275716	*D. glomerata* (Corda 1840) Qian Chen et L. Cai 2015
VKM F-2782	*A. quisqualis* Cesati 1852	PX289731	*-*	PX283755	PX289755	*D. pomorum* (Thümen 1879) Qian Chen et L. Cai 2015
VKM F-5158	*Ampelomyces quercinus* (Sydow 1915) Rudakov 2015	PX275618	PX283774	PX283762	PX275714	*Nothophoma brennandiae* Hernández-Restrepo, L.W. Hou, L. Cai et P.W. Crous 2020
VKM F-5159	*Ampelomyces quercinus* (Sydow 1915) Rudakov 2015	PX275619	PX283775	PX283763	PX275715	*N. quercina* (Sydow et P. Sydow 1915) Qian Chen et L. Cai 2015
VKM F-5161	*Ampelomyces* sp.	PX275621	PX283777	PX283765	PX275717	*N. spiraeae* L.X. Zhang et X.L. Fan 2020

**Table 4:** Phenotypic characteristics of the reference strains.

Strain Number (T or R Culture)	Species	Colony Diameter (cm) After 7 Days on Media			Colony Colours on OA		Size (μm)		Reference
		OA	PDA	MEA	Mycelium	Reverse	Pycnidia	Conidia	
CBS 528.66	*Didymella* *glomerata*	3.5–7.0	-	6.5–7.5	olivaceous	olivaceous to blackish	100–300	(3.5)4–8.5(10) × 1.5–3(3.5)	[27]
CBS 539.66	*D. pomorum*	4.5–6.0	-	5.5–7.5	olivaceous	brownish to blackish	100–200	(4)5–7(8) × 1.5–2.5(3)	[27]
CBS 145912	*Nothophoma* *brennandiae*	5.0–5.5	5.0–5.5	4.7–5.0	dark brick to sepia, cinnamon to the edge	-	155–350 × 100–300	3–8.5 × 1.5–3	[28]
CBS 633.92	*N. quercina*	5.5–6.8	-	5.5–7.5	olivaceous near the colony centre	concolourous	(50–)65–130(–150) × (65–)95–200(–220)	(5–)5.5–7.5(–8.5) × 3–4.5(–5)	[29]
CFCC 53928	*N. spiraeae*	-	-	-	hazel	-	(145–)155–280(300) × (120–)140–230(–250)	5–6.5(–7) × (3)3.5–4	[30]

## Data Availability

The DNA sequences obtained in this study have been submitted to GenBank.

## Supplementary Materials

The following supporting information can be downloaded at https://www.mdpi.com/article/10.3390/jof12080558/s1. Table S1: GenBank accession numbers of reference strains; Table S2: Identity (%) of studied strains with reference sequences of *Ampelomyces* spp. in NCBI.
