# Supplementary material for: Taxonomic Revision of Ampelomyces Strains (Dothideomycetes, Pleosporales) from All-Russian Collection of Microorganisms
Source: J Fungi (Basel). 2026 Jul 30;12(8):558. doi: 10.3390/jof12080558 (PMC13514647; doi:10.3390/jof12080558)
Supplement: Supplementary file 1 [file jof-12-00558-s001.zip › Table S1.pdf]

Table S1. GenBank accession numbers of reference strains.

Fungal species: this study	Strain number	Status	GenBank accession numbers			
			ITS	LSU	rpb2	tub2
Didymella aeria	CGMCC 3.18353	T	KY742051	KY742205	KY742137	KY742293
D. aurea	CBS 269.93	T	GU237818	GU237999	KT389599	GU237557
D. coffeae-arabicae	CBS 123380	T	FJ426993	GU238005	KT389603	FJ427104
D. combreti	CBS 137982	T	MN973525	MN943731	MT018139	MT005626
D. curtisii	CBS 251.92	R	FJ427038	GU238013	MT018131	FJ427148
D. eucalyptica	CBS 377.91	R	GU237846	GU238007	KT389605	GU237562
D. glomerata	CBS 528.66	R	FJ427013	EU754184	GU371781	FJ427124
D. glomerata	CBS 287.76	T	FJ427006	MH872750	MN983461	FJ427117
D. hippuris	YMF1.05089	T	MH257388	MH257473	MH311827	MH423015
D. keratinophila	CBS 143032	T	LT592901	LN907343	LT593039	LT592970
D. magnoliae	MFLUCC 18-1560	T	MK347814	MK348033	MK434852	—
D. pomorum	CBS 285.76	—	FJ427053	GU238025	KT389615	FJ427163
D. pomorum	CBS 286.76	—	FJ427054	GU238026	MT018128	FJ427164
D. pomorum	CBS 539.66	R	FJ427056	GU238028	KT389618	FJ427166
D. sancta	CBS 281.83	T	FJ427063	GU238030	MT018132	MT005619
D. sinensis	CGMCC 3.18348	T	KY742085	KY742239	MT018127	KY742327
D. tabebuiicola	COAD3340	T	MZ703618	MZ703623	MZ712360	MZ712364
Nothophoma brennandiae	CBS 140541	—	MN973558	MN943765	MT018202	MT005661
N. brennandiae	CBS 140540	—	MN973557	MN943764	MT018201	MT005660
N. brennandiae	CBS 140542	—	MN973556	MN943763	MT018200	MT005659
N. brennandiae	CBS 125539	—	MN973553	MN943760	MT018197	MT005656
N. brennandiae	CBS 125540	—	MN973555	MN943762	MT018199	MT005658
N. brennandiae	MF-32.68	—	MH651540	MH651581	PP750312	PP750331
N. brennandiae	CBS 262.95	—	MN973554	MN943761	MT018198	MT005657
N. brennandiae	CBS 159.37	—	MN973004	MN973393	—	MN984016
N. brennandiae	JW 1066	—	MN823578	MN823429	MN824603	MN824752
N. brennandiae	JW 53011	T	MN823579	MN823430	MN824604	MN824753
N. ferruginea	G:00127285	T	KY964330	KY964331	—	—
N. juglandis	CGMCC3.23563	—	MZ519403	—	MZ502664	MZ570583
N. juglandis	CGMCC3.23564	—	MZ519404	—	MZ502665	MZ570584
N. juglandis	CGMCC3.23565	—	MZ519405	—	MZ502666	MZ570585
N. platycladus	CGMCC3.27071	—	PP600103	—	PP595979	PP595986
N. platycladus	CGMCC3.27069	—	PP600101	—	PP595977	PP595984
N. platycladus	CGMCC3.27070	—	PP600102	—	PP595978	PP595985
N. populi	1-11	—	PQ462360	—	PQ473514	PQ473517
N. populi	1-10	—	PQ462358	—	PQ473512	PQ473515
N. populi	2-31	—	PQ462359	—	PQ473513	PQ473516
N. pruni	MFLUCC 18-1601	—	MH827005	MH827026	MH853662	MH853669
N. pruni	JZB380017	—	MH827006	MH827027	MH853663	MH853670
N. pruni	JZB380015	—	MH827004	MH827025	MH853661	MH853668
N. pruni	MFLUCC 18-1600	T	MH827007	MH827028	MH853664	MH853671
N. spiraeae	CFCC 53928	—	MN737833	MN737828	MN879292	MN879295
N. spiraeae	CFCC 53929	—	MN737834	MN737829	MN879293	MN879296
Phoma herbarum	CBS 615.75	T	KF251212	KF251715	KP330420	FJ427133
