# Supplementary material for: Taxonomic Revision of Ampelomyces Strains (Dothideomycetes, Pleosporales) from All-Russian Collection of Microorganisms
Source: J Fungi (Basel). 2026 Jul 30;12(8):558. doi: 10.3390/jof12080558 (PMC13514647; doi:10.3390/jof12080558)
Supplement: Supplementary file 1 [file jof-12-00558-s001.zip › Table S2.pdf]

Table S2. Identity (%) of studied strains with reference sequences of *Ampelomyces* spp. in NCBI.

Taxa	Representative strain No.	DNA-marker	Reference sequence in NCBI	VKM strains in this study (number of strains and number of sequences)											
				**F-2768**	**F-2794**	**F-2797**	**F-2799**	**F-2800**	**F-2839**	**F-5157**	**F-5160**	**F-2782**	**F-5158**	**F-5159**	**F-5161**
				**PX289730**	**PX289732**	**PX289733**	**PX289734**	**PX289735**	**PX289736**	**PX275617**	**PX275620**	**PX289731**	**PX275618**	**PX275619**	**PX275621**
Ampelomyces quisqualis	CBS 128.79	ITS	**HQ108037**	86.74	86.74	86.74	87.02	86.74	86.74	86.74	86.74	86.67	86.50	86.23	86.50
A. quisqualis	CBS 128.79	ITS	**MH861185**	84.57	84.57	84.57	84.78	84.57	84.57	84.47	84.57	84.50	84.52	84.18	84.39
A. quisqualis	CBS 129.79	ITS	**MH861186**	84.57	84.57	84.57	84.78	84.57	84.57	84.47	84.47	84.50	84.52	84.18	84.39
A. quisqualis	CBS 129.79	ITS	**KY090653**	86.74	86.74	86.74	87.02	86.74	86.74	86.74	86.74	86.67	86.50	86.23	86.50
A. quisqualis	CBS 129.79	ITS	**HQ108038**	86.74	86.74	86.74	87.02	86.74	86.74	86.74	86.74	86.67	86.50	86.23	86.50
A. quisqualis	CBS 130.79	ITS	**U82449**	83.30	83.30	83.30	83.52	83.30	83.30	83.30	83.30	83.22	83.11	82.88	83.11
A. quisqualis	CBS 130.79	ITS	**HQ108039**	86.74	86.74	86.74	87.02	86.74	86.74	86.74	86.74	86.67	86.50	86.23	86.50
A. quisqualis	CBS 130.79	ITS	**MH861187**	84.43	84.43	84.43	84.65	84.43	84.43	84.43	84.43	84.37	84.26	84.04	84.26
A. quisqualis	CBS 131.79	ITS	**MH861188**	84.43	84.43	84.43	84.65	84.43	84.43	84.43	84.43	84.37	84.26	84.04	84.26
A. quisqualis	CBS 131.79	ITS	**HM124975**	83.74	83.74	83.74	83.96	83.74	83.74	83.74	83.74	83.67	83.56	83.33	83.56
A. quisqualis	CBS 131.79	ITS	**HQ108040**	86.74	86.74	86.74	87.02	86.74	86.74	86.74	86.74	86.67	86.50	86.23	86.50
A. quisqualis	CBS 131.31	ITS	**AF035781**	84.65	84.65	84.65	84.43	84.65	84.65	84.55	84.65	84.43	84.75	84.68	84.68
A. quisqualis	CBS 133.32	ITS	**JN417758**	92.08	92.08	92.08	92.08	92.08	92.08	92.08	92.08	91.63	92.08	92.08	92.08
A. quisqualis	CBS 133.32	ITS	**JX681067**	86.51	86.51	86.51	86.54	86.51	86.51	86.42	86.51	86.08	86.53	86.32	86.32
A. quisqualis	ATCC 200246	ITS	**HQ108030**	92.08	92.08	92.08	92.08	92.08	92.08	92.08	92.08	91.63	92.08	92.08	92.08
A. quisqualis	ATCC 200247	ITS	**HQ108031**	91.70	91.70	91.70	91.70	91.70	91.70	91.70	91.70	91.25	91.70	91.70	91.70
Ampelomyces sp.	ATCC 200248	ITS	**HQ108032**	92.08	92.08	92.08	92.08	92.08	92.08	92.08	92.08	91.63	92.08	92.08	92.08
Ampelomyces sp.	ATCC 200249	ITS	**HQ108033**	92.45	92.45	92.45	92.45	92.45	92.45	92.02	92.45	92.45	92.45	92.45	92.45
Ampelomyces sp.	ATCC 200250	ITS	**HQ108034**	92.08	92.08	92.08	92.08	92.08	92.08	92.08	92.08	91.67	92.08	92.08	92.08
Ampelomyces sp.	ATCC 201056	ITS	**AF035780**	86.08	86.08	86.08	86.11	86.08	86.08	85.99	86.08	85.65	86.11	85.90	85.90
Ampelomyces sp.	ATCC 201059	ITS	**U82452**	95.76	95.72	95.73	95.56	95.73	95.74	95.53	95.53	96.10	95.56	95.34	95.53
Ampelomyces sp.	DSM 4624	ITS	**HQ108041**	90.75	90.75	90.75	90.75	90.75	90.75	90.75	90.75	90.83	90.75	90.75	90.75
Ampelomyces sp.	DSM 2225	ITS	**HQ108042**	86.51	86.51	86.51	86.54	86.51	86.51	86.42	86.51	86.08	86.53	86.32	86.32

Ampelomyces sp.	DSM 2222	ITS	**U82450**	87.93	87.93	87.93	87.96	87.93	87.96	87.93	87.93	87.40	87.70	87.70	87.70
Ampelomyces sp.	DSM 2223	ITS	**U82451**	84.24	84.24	84.24	84.24	84.24	84.24	84.32	84.24	84.04	84.50	84.31	84.28

**Taxa**	**Representati ve strain No.**	**DNA-marker**	**Reference sequence in NCBI**	**VKM F- strains in this study (number of strains and number of sequences)**											
				**2768**	**2794**	**2797**	**2799**	**2800**	**2839**	**5157**	**5160**	**2782**	**5158**	**5159**	**5161**
				**PX289754**	**PX289756**	**PX289757**	**PX289758**	**PX289759**	**PX289760**	**PX275713**	**PX275716**	**PX289755**	**PX275714**	**PX275715**	**PX275717**
Ampelomyces quisqualis	CBS 128.79	LSU	**MH872954**	95.12	95.12	95.40	95.44	95.40	95.34	94.45	94.41	94.99	95.34	95.05	95.34
A. quisqualis	CBS 128.79	LSU	**JX681063**	95.12	95.12	95.40	95.44	95.40	95.34	94.45	94.41	94.99	95.34	95.05	95.34
A. quisqualis	CBS 129.79	LSU	**MH872955**	95.10	95.10	95.38	95.43	95.38	95.32	95.43	95.41	94.97	95.32	95.05	95.32
A. quisqualis	CBS 131.79	LSU	**JX681065**	95.26	95.26	95.52	95.56	95.52	95.47	95.49	95.41	95.13	95.39	95.05	95.38
A. quisqualis	CBS 131.31	LSU	**MH866605**	94.84	94.84	95.13	95.18	95.13	95.07	95.19	95.18	94.71	94.96	95.06	94.96
A. quisqualis	CBS 131.31	LSU	**JX681066**	95. 26	95. 26	95. 52	95. 57	95.27	95.47	95.49	95.42	95. 14	95.28	95.06	95.27
A. quisqualis	CBS 133.32	LSU	**MH866692**	94.90	94.90	95.18	95.23	95.18	95.12	95.14	95.06	94.78	94.93	94.68	94.92
Ampelomyces sp.	ATCC 200245	LSU	**AY293793**	93.71	93.71	93.71	93.71	93.71	93.71	93.55	93.42	93.56	93.43	93.56	93.41
Ampelomyces sp.	ATCC 201056	LSU	**AY293794**	93.43	93.43	93.43	93.43	93.43	93.43	93.27	93.14	93.29	93.01	92.52	92.99
